# Improved circRNA Identification by Combining Prediction Algorithms

**DOI:** 10.3389/fcell.2018.00020

**Published:** 2018-03-05

**Authors:** Thomas B. Hansen

**Affiliations:** Department of Molecular Biology and Genetics and Interdisciplinary Nanoscience Center, Aarhus University, Aarhus, Denmark

**Keywords:** non-coding RNA, circular RNA, gene prediction, bioinformatics, combining algorithms

## Abstract

Non-coding RNA is an interesting class of gene regulators with diverse functionalities. One large subgroup of non-coding RNAs is the recently discovered class of circular RNAs (circRNAs). CircRNAs are conserved and expressed in a tissue and developmental specific manner, although for the vast majority, the functional relevance remains unclear. To identify and quantify circRNAs expression, several bioinformatic pipelines have been developed to assess the catalog of circRNAs in any given total RNA sequencing dataset. We recently compared five different algorithms for circRNA detection, but here this analysis is extended to 11 algorithms. By comparing the number of circRNAs discovered and their respective sensitivity to RNaseR digestion, the sensitivity and specificity of each algorithm are evaluated. Moreover, the ability to predict *de novo* circRNA, i.e., circRNAs not derived from annotated splice sites, is also determined as well as the effect of eliminating low quality and adaptor-containing reads prior to circRNA prediction. Finally, and most importantly, all possible pair-wise combinations of algorithms are tested and guidelines for algorithm complementarity are provided. Conclusively, the algorithms mostly agree on highly expressed circRNAs, however, in many cases, algorithm-specific false positives with high read counts are predicted, which is resolved by using the shared output from two (or more) algorithms.

## Introduction

Circular RNA (circRNA) is now a well-established class of non-coding RNA defined by a covalently closed circular structure facilitated by non-linear splicing (backsplicing). The first functionally characterized circRNA, CDR1as/ciRS-7, was shown to be highly abundant in neurons and to act as a miR-7 regulator or sponge using the 70+ encoded miR-7 binding sites (Hansen et al., 2013; Memczak et al., 2013). CDR1as/ciRS-7 is itself post-transcriptionally regulated by miR-671-mediated endo-cleavage (Hansen et al., 2011), and just recently, CDR1as/ciRS-7 was shown to have neurological phenotypes in knock-out mice (Piwecka et al., 2017). Many circRNAs have now been ascribed miRNA sponge functionalities, although the biological relevance and the evolutionary significance has still not been convincingly established. In addition, a subset of circRNAs has been shown to contain circRNA-specific ORFs and to encode proteins (Legnini et al., 2017; Pamudurti et al., 2017; Yang et al., 2017).

While the functions of most circRNAs are still elusive, great progress has so far been done toward profiling and characterizing the landscape and dynamic expression of circRNAs in various cell lines, tissues, organisms, and in disease (Salzman et al., 2013; Rybak-Wolf et al., 2015). Here, the setup typically relies on Ribosome-depleted RNA subjected to high throughput sequencing. The resulting sequence reads are then analyzed using one of many developed algorithms dedicated to identify and quantify the expression of circRNAs specifically. The reliability of the output is obviously crucial for proper annotation of the circRNA landscape in the given samples. Recently, a thorough description and comparison of circRNA pipelines was published (Zeng et al., 2017), and we previously analyzed and compared the output from 5 different algorithms (Hansen et al., 2015): circRNA_finder (Westholm et al., 2014), CIRCexplorer (Zhang et al., 2014), CIRI (Gao et al., 2015), find_circ (Memczak et al., 2013), and MapSplice (Wang et al., 2010). Here, this analysis is extended by 6 additional algorithms: ACSF (You et al., 2015), CIRCexplorer2 (Zhang et al., 2016), CIRI2 (Gao et al., 2017), DCC (Cheng et al., 2016), KNIFE (Szabo et al., 2015), and Uroborus (Song et al., 2016) (See Table 1). Overall, the sensitivity and specificity of predicted circRNAs are compared by assessing the quantities of circRNAs found by each algorithm as well as the fraction of true and false positives in the output—judged by RNAse R resistance. The impact on pre-processed reads, i.e., low quality read removal and adaptor-trimming, on the performance of each algorithm is also addressed. In addition, the *de novo* prediction (without relying on gene annotation) accuracy of the algorithms capable of annotation-independent circRNA prediction are evaluated, and finally, the gain of conjoining the output from any two algorithms is evaluated, which shows that all algorithms, although to varying degrees, benefit from combining and merging the circRNA prediction output with other algorithms.

**Table 1:** Algorithms.

**Tool**	**Version**	**Language**	**Mapper**	***De novo*?**	**URL**
ACFS	2.1	Perl	Bwa	Yes	https://github.com/arthuryxt/acfs
CIRCexplorer	1.0.6	Python	Tophat	No	https://github.com/YangLab/CIRCexplorer
CIRCexplorer2	2.0.1	Python	Tophat	Yes	https://github.com/YangLab/CIRCexplorer2
circRNA_finder	N/A	Perl	STAR	Default	https://github.com/orzechoj/circRNA_finder
CIRI	1.2	Perl	Bwa	Default	https://sourceforge.net/projects/ciri/files
CIRI2	2.0.6	Perl	Bwa	Default	https://sourceforge.net/projects/ciri/files/CIRI2/
DCC	0.4.4	Python	STAR	Default	https://github.com/dieterich-lab/DCC
Find_circ	N/A	Python	Bowtie2	Default	http://www.circbase.org/
KNIFE	1.4	Python/Perl/R	Bowtie2	Yes	https://github.com/lindaszabo/KNIFE
Mapsplice	2.1.8	Python	Tophat	No	http://www.netlab.uky.edu/p/bioinfo/MapSplice2
Uroborus	0.1.2	Perl	Tophat	No	https://github.com/WGLab/UROBORUS

## Materials and methods

### Prediction of circRNA

Prediction of circRNA was performed as described previously (Hansen et al., 2015): Briefly, RNA sequencing (RNAseq) samples (see Supplementary Figure 1A) were downloaded from the Sequence Reads Archive (SRA). Processing of reads was performed with trim-galore using the following parameters: -f fastq -e 0.1 -q 20 -O 1 -a AGATCGGAAGAGC. For each RNAseq sample, circRNA prediction was conducted with 11 different algorithms (see Table 1) adhering to the default settings by the respective authors and using the GRCh37 (hg19) genome assembly. Gene-annotations were collected from UCSC genome browser (UCSC Genes track), iGenomes (*hg19.ref.gtf*), and Ensembl (*Homo_sapiens.GRCh37.66.gtf*). The scripts and settings used are outlined in *bash.sh* (available in the Supplementary Zip-file).

### Analysis of prediction

For each algorithm, circRNA prediction was performed separately on all samples (Supplementary Figure 1) and subsequently the output was merged with custom python scripts (available upon request) into BED files (see Supplementary Zip-file). For each algorithm, only circRNAs with at least three reads in one of the untreated samples were kept for analysis. For KNIFE, additionally, circRNAs with a posterior probability below 0.9 were discarded, and for CIRCexplorer, the circular intronic RNA (ciRNA) candidates were omitted in the subsequent analysis. Furthermore, in contrast to our previous analyses, all chrM-derived candidates were collectively removed. The sum of reads spanning the backsplice junction in the two control samples was used as a measure of expression level. For each algorithm, circRNAs were classified as RNaseR resistant or RNaseR sensitive if a 5-fold enrichment or a reduction in the RNAseR treated samples was observed, respectively, however for the samples from Mercer et al. (2015) 2- and 0.7-fold cutoffs were used. Furthermore, to enable comparison between algorithms, the starting coordinate was converted to 0-based for certain algorithms (ACFS, circRNA_finder, CIRI, CIRI2, DCC, and MapSplice). For *de novo* prediction, the algorithms with mandatory gene-annotation input (CIRCexplorer2, KNIFE and MapSplice) were provided a mock annotation file to eliminate any annotation-based predictions.

### Annotation of circRNAs

To estimate the mature length of circRNAs, annotated mRNAs from UCSC genes track utilizing the splice sites involved in backsplicing were used as templates for circRNA exon-intron structure, i.e., the circRNAs were assumed to have similar internal splicing pattern as the corresponding host gene. In case of multiple overlapping isoforms, the average of deduced lengths was used.

### Availability

The merged output from each algorithm is available in the Supplementary Zip file. Moreover, comparison of algorithms can be studied interactively at www.ncrnalab.dk/battle_of_algorithms.

## Results

### RNAse R resistance

**Figure 1:**
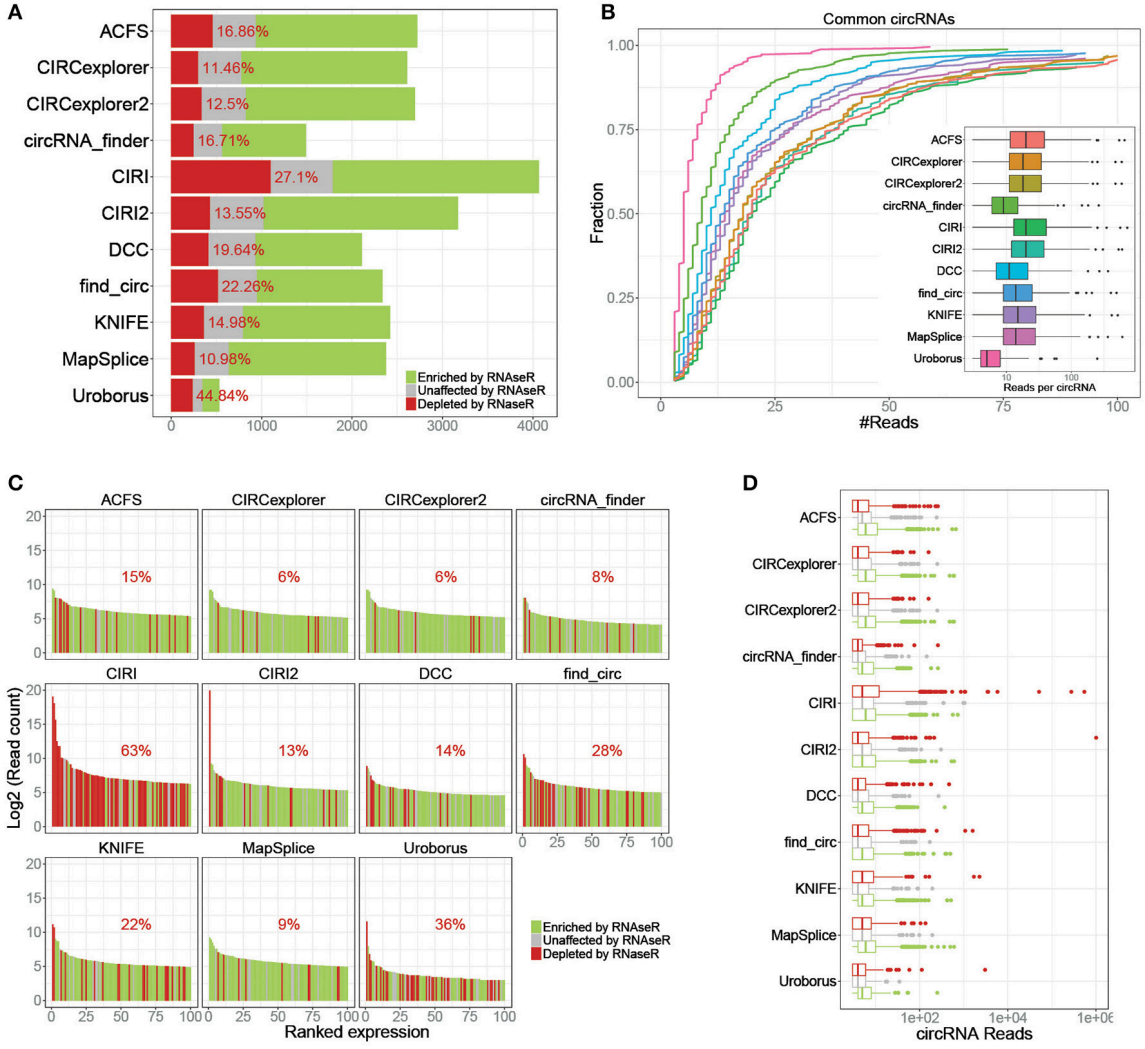
Specificity and sensitivity. **(A)** Stacked barplot of all predicted circRNAs stratified by RNAse R resistant (≥ 5 fold enrichment, green), unaffected (1–5 fold enrichment, gray) and RNAse R sensitive (depleted in RNaseR treated samples, red), as denoted. Percentage reflects the fraction of RNaseR sensitive circRNAs defined as false positives. **(B)** Cumulative fraction plot of read-counts for circRNAs shared by all 11 algorithms (*n* = 259) color coded as denoted in the associated boxplot where reads per circRNA is shown. **(C)** Ranked plot of the top 100 expressed circRNAs predicted by each algorithm color-coded as in **A**. Percentage reflects the fraction of RNase R sensitive circRNAs (false positives) within the plotted top 100. **(D)** Boxplot of circRNA expression predicted by each algorithm stratified by RNaseR sensitivity (as in **A**).

Similar to our previous comparison of circRNA prediction algorithms (Hansen et al., 2015), we used a deep and comprehensive dataset on untreated (SRR444655 and SRR444975) and RNAseR treated (SRR444974 and SRR445016) samples (Jeck et al., 2013). Here, circRNAs were predicted in all 4 samples using 11 available algorithms, and based on the circRNA output from the untreated samples, the enrichment of each circRNA in the RNAseR treated samples was determined. Then, for each algorithm the fraction of RNAseR depleted, unchanged and enriched putative circRNA was plotted (Figure 1A) showing between 500 and 4,000 putative circRNAs of which 11–47% are depleted by RNAseR indicating a remarkable difference between algorithm output. Overall, the performance of the algorithms should be measured by specificity and sensitivity. Here, specificity is determined by lack of false positives, i.e., RNAse R sensitive species, and here, CIRCexplorer (1 and 2) and MapSplice outperform the others by predicting only ~11% RNAseR sensitive species. Conversely, analyzing the circRNA subset commonly predicted by all algorithms (*n* = 259), the sensitivity is here defined by the number of backsplice-spanning reads found by each algorithm. Here, most algorithms have median expression of 14–20 reads, while DCC, circRNA_finder, and Uroborus show the lowest sensitivity with 11, 9, and 5 median reads per circRNA, respectively (Figure 1B).

While sensitivity and the overall specificity are important aspects of predicting algorithms, the lowest expressed species are typically filtered out in downstream analyses. Thus, focusing specifically on the top 100 expressed circRNAs predicted by each algorithm (Figure 1C), we largely observe an increased specificity for all algorithms except for CIRI, where 63% of the top100 expressed circRNAs are RNaseR sensitive. In fact, plotting the expressing of all predicted circRNAs stratified by algorithm and RNaseR sensitivity, the highest expressed circRNA candidates according to many algorithms are, alarmingly, RNaseR sensitive (Figure 1D). Overall, the 14 most highly expressed candidates are RNaseR depleted (8 of these are uniquely predicted by CIRI), and surprisingly, these seemingly false positives are not caused by one particular highly abundant locus, but are 14 distinct loci (Supplementary Table 1). Moreover, apart from one candidate (predicted by both find_circ and KNIFE), all are exotic circRNA, i.e. circRNA candidates only predicted by one algorithm. Setting aside the potential problems of high-scoring candidates with questionable validity, for all algorithms *bona fide* circRNAs are in general higher expressed than the false positives (Figure 1D).

### Raw vs. trimmed reads

**Figure 2:**
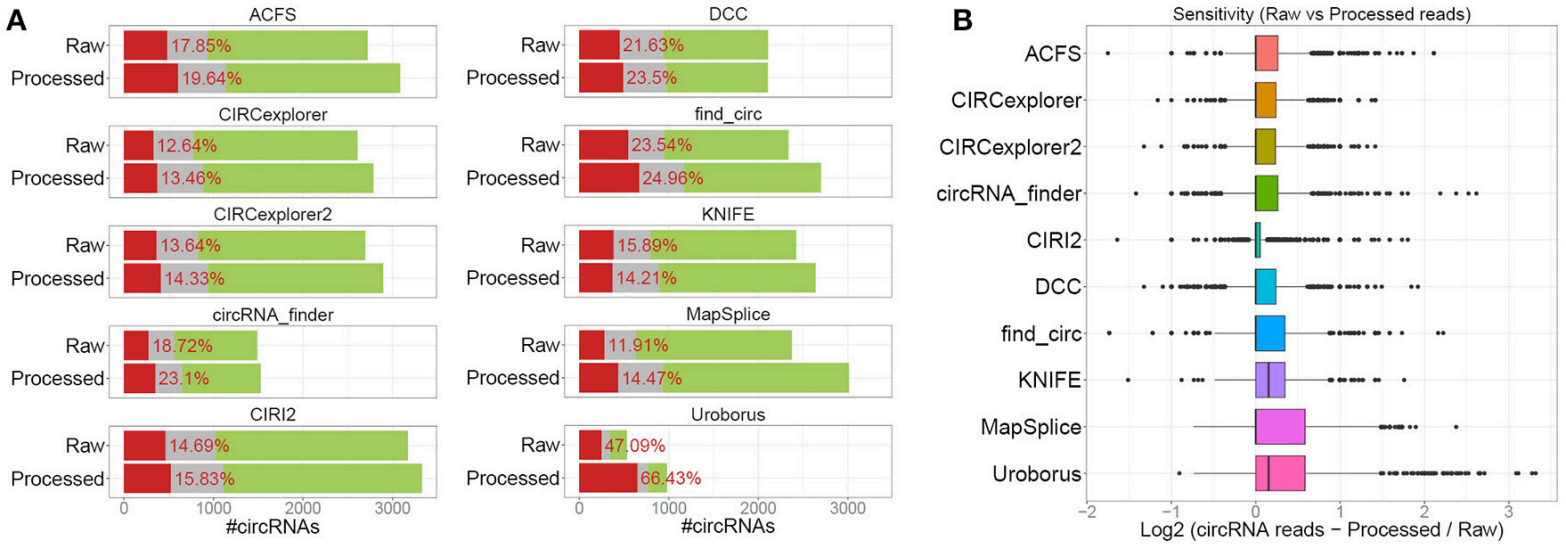
Raw vs. processed reads. **(A)** Stacked barplot as in Figure 1A comparing the output from raw (“Raw,” as seen in Figure 1A) and circRNA prediction using pre-processed reads (“Processed”). **(B)** Boxplot comparing the read-counts on circRNAs shared between “Raw” and “Processed” prediction.

In the above analysis, the raw sequence reads were used in the prediction. However, as the algorithms use different approaches to identify back-splice spanning reads, the impact of read quality and the partly presence of 3' adaptor sequences in a subset of reads were determined by re-conducting all predictions (except for CIRI that requires equal read-length between read pair) on pre-processed reads. Here, the overall output was very similar to the raw read analysis (Figure 2A), however, in general, the number of predicted circRNAs increased by 0–27% and most notably for Uroborus with almost twice as many (83%) circRNAs predicted. Surprisingly, this increase came with a concomitant increase in fraction of false positives (13–67%) compared to 12–48% using raw sequence reads. Moreover, the sensitivity measured by the read count on circRNAs found using both raw and processed reads were also modestly increased, however most algorithms showed unaffected median expression (Figure 2B). Certain algorithms benefit more from read trimming than others. Especially for KNIFE, processing the reads prior to running the algorithm seems beneficial whereas for other algorithms, such as circRNA_finder and Uroborus, preprocessing of reads actually reduced the quality of prediction output notably. It should be emphasized, that these observations are based on one particular high quality dataset with only a marginal set of reads being trimmed or removed during processing (Supplementary Figure 1B), and therefore the effects on circRNA prediction could be much more pronounced in other datasets with lower quality and increased prevalence of adaptor sequences.

### *De novo* prediction

**Figure 3:**
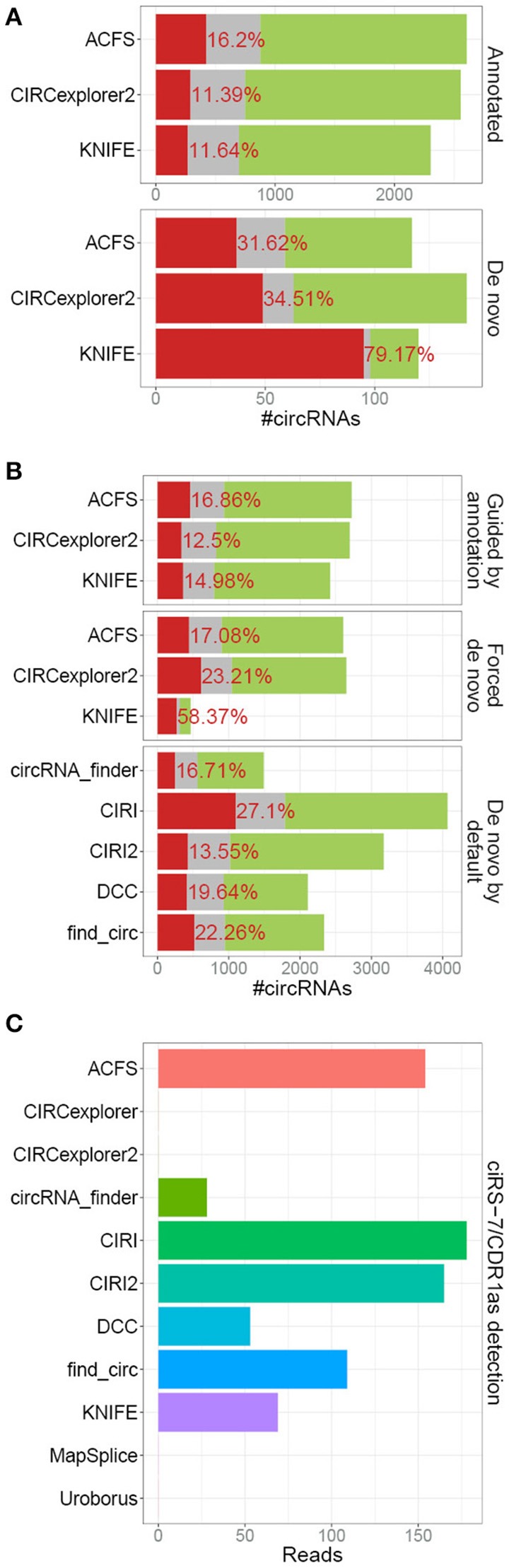
*De novo* prediction of circRNAs**. (A)** Stacked barplot comparing the annotated and un-annotated (*de novo*) default outputs from ACFS, CIRCexplorer2, and KNIFE. **(B)** Stacked barplot comparing overall circRNA predictions output from algorithms (ACFS, CIRCexplorer2, and KNIFE) either guided by annotation (default setting, as in Figure 1A) or when forced *de novo* using mock annotations with algorithms *de novo* by default (circRNA_finder, DCC, CIRI, CIRI2, and find_circ, as in Figure 1A). **(C)** Back-splice spanning read counts on ciRS-7 obtained from each algorithm as an example of *de novo* prediction. For KNIFE, the *de novo* resolution is 50 bp and ciRS-7 was here defined as chrX:139865300-139866900.

In our previous analyses, we compared the bulk output from 5 different algorithm of which 2 (CIRCexplorer and MapSplice) were assisted by exon annotation and thus by default only predicting circRNA derived from annotated splice sites, which comprises the vast majority of *bona fide* circRNA. This, obviously, provided these algorithms with information useful for demarcating circRNAs from background noise and the comparison was therefore not completely fair. CIRCexplorer, Uroborus, and MapSplice exclusively output circRNAs derived from annotated splice sites, whereas others, ACSF, CIRCexplorer2 and KNIFE, require mandatory gene annotation but they provide an additional *de novo* list of circRNAs. Comparing the RNAseR sensitivity of annotated circRNAs, i.e., circRNAs derived from annotated splice sites, with circRNAs derived from unannotated splice sites, the *de novo* circRNAs (comprising roughly 5% of total circRNA output), clearly shows that *de novo* predicted circRNAs are less likely to be true positives (Figure 3A). The high fraction of RNAseR sensitive candidates in the *de novo* subset of circRNAs is not necessarily a fair reflection of the *de novo* performance of these algorithms, as most bona fide circRNAs are associated with annotated splice sites. Instead, ACSF, CIRCexplorer2 and KNIFE were provided with mock annotations to force *de novo* predictions exclusively. Here, all three algorithms performed less effective, although only a small to modest difference between guided and *de novo* prediction was observed for ACSF and CIRCexplorer2 (Figure 3B). KNIFE, in contrast, performed dramatically inferior without annotation, suggesting that the *de novo* feature of KNIFE prediction is not very reliable. In addition, when comparing the forced *de novo* prediction with default *de novo* algorithms, CIRI2 serves as the most trustworthy predictor (Figure 3B).

One particular example of *de novo* prediction is the well-characterized circRNA, CDR1as/ciRS-7 (Hansen et al., 2011, 2013; Memczak et al., 2013; Piwecka et al., 2017). CiRS-7 is not derived from pre-annotated host transcript and consistently linear splicing to this exon is only detectable at very low levels. Here, all the algorithms with *de novo* prediction capabilities except for CIRCexplorer2 were able to predict ciRS-7 although with various sensitivities (Figure 3C). This limitation of annotation-guided algorithms can easily be overcome by manually annotating the small subset of bona fide circRNAs not deriving from annotated splice sites prior to prediction, however, this is only an option for well-annotated organisms, such as humans, and obviously not applicable to species with an uncharacterized circRNAome.

### Improving find_circ

Find_circ was one of the first pipelines available to disclose circRNA in ribo-depleted RNAseq, and probably one of the most widely used algorithms for circRNA detection to date. As seen above, find_circ is not the best performing algorithm available, and using default settings, one specific falsely annotated circRNA candidate is predicted as very abundant by find_circ solely (Supplementary Figure 2A). This circRNA candidate, derived from the TUBA1A-TUBA1B locus, was earlier recognized as an example of closely related tandem genes where conventional splicing was misinterpreted as backsplicing between the neighboring genes (Gao et al., 2015). Find_circ outputs the mapping quality (i.e., the probability of misaligned read) of the mapped anchor sequences, and from this, it is evident that the TUBA1A-TUBA1B candidate has a suboptimal mapping quality, indicating as expected that the reads supporting TUBA1A-TUBA1B backsplicing are genomic multimappers. However, increasing the threshold for mapping quality from one anchor with ≥ 35 (default setting) to both anchors having the highest possible quality, i.e., 40, not only completely removes the TUBA1A-TUBA1B mis-annotation (Supplementary Figure 2A) but also reduces the fraction of RNaseR sensitive circRNAs from 23 to 15% (Supplementary Figure 2B). Moreover, additional abundant but falsely annotated species are discarded by the increased stringency (Supplementary Figure 2C). Although as expected, this comes with a concomitant but modest decrease in overall numbers of predicted circRNAs (Supplementary Figure 2B). Therefore, if find_circ is the algorithm of choice for circRNA prediction, it is highly recommendable to increase the mapping quality threshold.

### False negatives

Global transcriptome analysis on RNaseR treated samples is not commonly performed, and to our knowledge the dataset from Jeck et al. (2013) currently constitutes the most extensive RNA sequencing performed on RNaseR treated samples (300–400 mio reads per sample, Supplementary Figure 1A). Although surprisingly, as also pointed out by Jeck et al, ciRS-7/CDR1as, a well-established circRNA, exhibits sensitivity toward RNaseR (Supplementary Figure 3A). This either suggests that: (i) ciRS-7, in contrast to many other experiments, is not a *bona fide* circRNA, at least in Hs68 cells. (ii) ciRS-7 has been nicked by miR-671 and therefore sensitive toward the RNaseR exonuclease. (iii) RNaseR has unspecific endonuclease activity and thus a subset of depleted species are in fact false negatives. With 1,485 nt mature length, ciRS-7 is one of the larger circRNAs known (circRNA median length is 555 nt, Supplementary Figure 3B). In fact, there is a significant correlation between circRNA mature length and RNaseR sensitivity (Supplementary Figure 3C) and the subset of RNaseR sensitive species commonly predicted by all 11 algorithms are in general much longer than the resistant subset (Supplementary Figure 3D). Assuming that these longer circRNA species are not false positives, this indicates that long circRNAs are more prone to unspecific RNaseR decay, and therefore validation and characterization of these species should be conducted with additional care.

### Combining prediction algorithms

**Figure 4:**
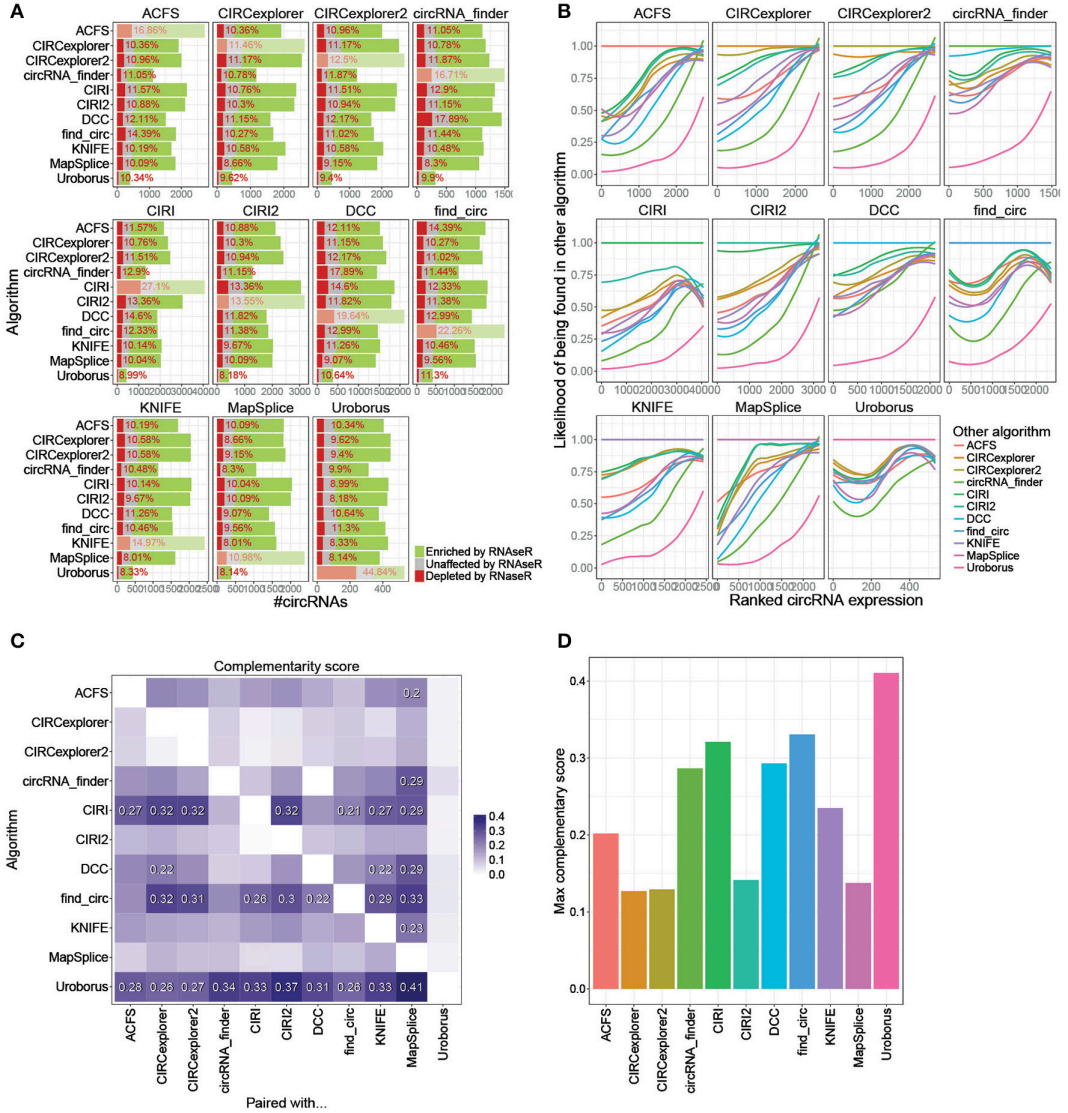
Conjoining algorithms**. (A)** Comprehensive stacked barplot analysis of RNaseR sensitivity in the shared predictions by any two algorithms. The “dimmed” bars denote the unpaired algorithm (as also seen in Figure 1A). **(B)** Loess regression on fraction of circRNAs found by other algorithm (color-coded as seen in the legend) as a function of ranked expression of circRNAs identified and quantified by algorithm denoted in strip. **(C)** Heatmap on Complementary score. The Complementary score is calculated as (iTF × iTN)^∧^2, where iTF is the fraction of true positive circRNAs (RNaseR resistant circRNAs) found in algorithm denoted on the y-axis and shared with algorithm on x-axis (Supplementary Figure 6A), and iTN is 1-fN, where fN is the fraction of RNaseR sensitive species conjointly identified in other algorithm (see Supplementary Figure 6B). Complementary scores ≥ 0.2 are denoted specifically. **(D)** For each algorithm, the maximum Complementary score (from **C**) is depicted.

Previously, we recommended that circRNA pipelines should be combined in order to avoid annotation of false positives (Hansen et al., 2015). Similarly, the common pair-wise output from all possible combinations of algorithms is here evaluated (Figure 4A, Supplementary Figure 4, and http://www.ncrnalab.dk/battle_of_algorithms). For most algorithms, combining the prediction with any other algorithm reduces the fraction of RNaseR sensitive candidate species from > 15% to around 10%. The algorithms tend to agree on the highly abundant - and presumably most relevant—circRNAs while the circRNAs discarded by combining two algorithms were typically of low abundance shown by a general high frequency of overlap between high-ranked circRNAs (Figure 4B). Basically, there is a clear selection of highly abundant and RNaseR resistant circRNA species when using the conjoined output from 5+ or 10+ algorithms compared to the exotic species unique to one algorithm (Supplementary Figures 5A,B), and while the overall bulk of predicted species is reduced upon merging algorithms, the omitted candidates are mostly false positives or lowly expressed circRNAs (Supplementary Figures 5B,C). In other words, most algorithms agree largely on the highly abundant *bona fide* circRNAs, and consequently, the inevitable out-filtering of circRNAs by selecting shared circRNAs predicted by two (or more) algorithms are almost exclusively species of low relevance.

While combining algorithms is generally a recommended approach, the specific pair of algorithms used is not irrelevant. For instance, predictions by circRNA_finder and DCC are both based on STAR-mapped reads, and therefore, presumably, these two algorithms seem less suitable to complement each other in terms of reducing the fraction of false positives (Figure 4A). To establish general guidelines for combination of algorithms, a rough measurement of complementarity was established: First, for each algorithm, an index of true positives (iTP), reflecting the fraction of preserved circRNAs with RNaseR resistance after conjoining with any other algorithm, was determined (Supplementary Figure 6A). For instance, CIRI outputs 2289 RNaseR resistant species of which 1551 are shared with ACSF resulting in an iTP of 0.68 (= 1551/2289). Then, similarly, the fraction of discarded true negatives (iTN, Supplementary Figure 6B) was calculated, which is the inverse fraction of shared true negatives. As an example, CIRI predicts 1105 RNaseR sensitive species where 251 are also found by ACSF and thus the iTN index is 0.77 (= 1–(251/1105)). Finally, to evaluate the overall conjoining effect of any two algorithms, a Complementary score is proposed (Complementary score = (iTPxiTN)^∧^2) that scores the achieved benefit of pairing one algorithm with any other algorithm (Figure 4C), e.g., the effect of pairing CIRI with ACSF is 0.27. Based on this it is evident that CIRI, find_circ, and Uroborus profit the most from combination with almost any other algorithm, while MapSplice seems to be the preferred complement to most algorithms. In addition, CIRI2, MapSplice, and the CIRCexplorers perform well single-handedly and here the combination with other algorithm only results in subtle improvements (Figures 4C,D). However, particularly seen here for CIRI2, while the overall performance is high, relying on one algorithm solely has the intrinsic risk of highly abundant exotic candidates being mis-annotated as bona fide circRNAs.

To determine the add-on effect of including a third algorithm, three-wise Complementary scores for all possible combinations were computed (see Supplementary Figure 6C). In general, there is a slight additional improvement when combining with a third algorithm, especially if the first two algorithms are DCC and circRNA_finder, which also showed lowest complementarity in the pair-wise comparison.

### Reproducibility

All of the above analyses are based on RNaseR treated and untreated samples from Jeck et al. (2013). The conclusions drawn could be dataset-specific and not general themes. To elucidate this, similar analyses were conducted on a subset of samples from Mercer et al. (2015) (see Supplementary Figure 7A, in our hands a few algorithms failed to finalize prediction on certain samples and these were then overall omitted). First, the fraction of RNaseR sensitive circRNAs predicted by each algorithm was compared (as in Figure 1A), however in contrast to the previous analysis, cutoffs for RNaseR resistance and RNaseR sensitivity were set at 2 and 0.7, respectively, to adjust for differences in library size and a less pronounced effect of RNaseR treatment on backsplice-spanning read-count. Here, once again the best performing algorithms are MapSplice, the CIRCexplorers and CIRI2, where CIRI2, as mentioned, is exclusively performing *de novo* predictions (Supplementary Figure 7B). Also, the pairwise combination of algorithms roughly reproduce the effect on prediction output (Supplementary Figures 7C,D). In particular, all algorithms benefit notably (Complementary score ≥ 0.2) from pairing with MapSplice or CIRI2. And, as seen above, CIRI, find_circ and Uroborus benefit from the combination with almost all other algorithms. This is here also seen for DCC and circRNA_finder, which is a reflection of a rather high level of false positives in their stand-alone prediction. Overall, this validates the general conclusions drawn from the initial analyses, and thus using shared output from two (or more) circRNA prediction algorithms is highly recommended.

## Discussion

Circular RNA is a fascinating subclass of non-coding RNA, and great efforts are currently invested in characterizing the circRNA landscape in various cell lines, tissues, developmental stages, and organisms. To this end, global transcriptome analysis must be conducted to gain insights into the non-polyadenylated world of circRNAs. Going from raw sequencing reads to circRNA detection and quantification requires specialized pipelines and algorithms. Here, the circRNA output from 11 different algorithms are compared, and, as shown above, this reveals notable differences between algorithms. Ranging from 533 to 4,067 circRNAs of which 11–45% are alleged false positives, the choice of prediction algorithm will greatly affect the quality and quantity of predicted circRNAs. Removing low quality and adaptor containing reads prior to prediction only had a modest effect, although this likely reflects that the dataset used already is of high quality.

The gold-standard biochemical delineation of circRNA and linear RNA is by RNaseR treatment, which on a global scale enriches circRNA significantly. However, as discussed above, longer circRNAs seem to exhibit lower resistance than shorter species. Therefore, titration of RNAseR and alternative validation methods such as northern blotting or polyA-minus enrichment could be employed if an expected circRNA candidate has a long mature length (~1.5 kb or above) and shows partial RNaseR-sensitivity. In contrast, it is likely that certain linear RNA species, e.g., structured transcripts with high GC-content, will resist the RNaseR digestion. Consequently, the general recommendation is to address the circular nature of RNAs of interest by several means.

The most challenging aspect of circRNA detection is *de novo* prediction without prior knowledge of exon-intron annotations. Many algorithms perform *de novo* prediction by default, while others use annotations to assist the prediction. Generally, circRNAs derived from novel and un-annotated splice sites are less likely to be true positives, and thus annotation-based prediction is essentially more reliable. As expected, comparison of annotation-based output with forced *de novo* prediction shows reduced overall accuracy from 12–16% false positives to 17–58%, which is more similar to algorithms being *de novo* by default (13–27%). Here, impressively, it should be noted, that CIRI2, which is *de novo* by default, performs comparably to annotation-assisted algorithms, and therefore in this setup, CIRI2 is the most reliable algorithm available. However, predicting circRNAs in thoroughly annotated organisms, the CIRCexplorers or MapSplice are also suitable choices.

Here, using online available datasets from Jeck et al. (2013) and Mercer et al. (2015) we have compared the output from 11 different circRNA prediction algorithms. The quality of prediction seems to translate well between datasets; however, there could be certain scenarios where the conclusions drawn here do not apply. For instance, the impact of using RNA sequencing dataset with either longer or shorter reads or applying the algorithms to other reference genomes has not been assessed. Moreover, as discussed above, *do novo* prediction is definitely more challenging than annotation-dependent prediction, and consequently annotation-dependent algorithms are best suited for well-studied organisms such a humans, whereas *do novo* algorithms are recommended for more exploratory research. In any case, the combination of algorithms results in notable improvements of overall prediction reliability independently of the dataset in question, and therefore this most likely applies to all circRNA prediction scenarios.

The indexes used to determine the effect of combining algorithms (iTP, iTN, and Complementary score, Figures 4C,D and Supplementary Figure 6) could be biased by the overall number of circRNAs predicted. In fact, the iTP scores correlate negatively with number of circRNAs from 1st algorithm (y-axis) but positively with 2nd algorithm (Pearson's correlations:−0.35 and 0.87, respectively, *p* < 1e-3), whereas the iTN scores are conversely correlating with circRNA numbers (Pearson's correlations: 0.13 and −0.68, respectively, *p* < 1e-16 for the latter). The Complementary scores, however, only show a modest although significant correlation with number of circRNAs predicted (−0.29 and 0.26, respectively, *p* < 0.01), but when omitting Uroborus from this analysis, no significant correlation was observed (0.13 and −0.04, *p* > 0.19), and therefore conclusively the Complementary score is not influenced considerably by numbers of predicted candidates.

Comparing the circRNA predictions indicate that the differences between algorithms are mostly seen in their respective weaknesses, i.e., the subset of false positives. For that reason, the fundamental motivation to focus selectively on the shared prediction by two algorithms is to eliminate false positives while preserving the vast majority of true positive circRNAs. In addition, as noted above, a large fraction of putative circRNAs candidates with high abundance are actually RNaseR sensitive but, importantly, algorithm-specific. Consequently, these exotic peculiarities are consistently omitted using shared output from circRNA prediction pipelines. Therefore, conclusively, circRNA predictions are much more reliable when two or more prediction algorithms are combined, and the minimal loss of true positives are greatly outweighed by the removal of false positives.

## Author contributions

The author confirms being the sole contributor of this work and approved it for publication.

### Conflict of interest statement

The author declares that the research was conducted in the absence of any commercial or financial relationships that could be construed as a potential conflict of interest.
