# Supplementary material for: Improved circRNA Identification by Combining Prediction Algorithms
Source: Front Cell Dev Biol. 2018 Mar 5;6:20. doi: 10.3389/fcell.2018.00020 (PMC5844931; doi:10.3389/fcell.2018.00020)
Supplement: Supplementary file 1 [file Presentation1.pdf]

Supplementary Information:

Supplementary Figures 1-7
Supplementary Table 1

Supplementary Figure 1:

A

Accession	Samples	Reference	Total Reads
SRR444655	Hs68 - control#1	Jeck *et al* , 2012	314106316
SRR444974	Hs68 - RNaseR#1	Jeck *et al* , 2012	316611710
SRR444975	Hs68 - control#2	Jeck *et al* , 2012	412725466
SRR445016	Hs68 - RNAseR#2	Jeck *et al* , 2012	399844972

B

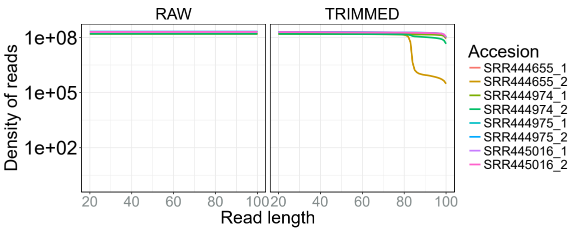

Supplementary Figure 1: The RNAseq dataset. A) Table with accession numbers, sample content and corresponding read counts. **B)** Density of read-lengths before (Raw) and after processing by trim_galore.

Supplementary Figure 2:

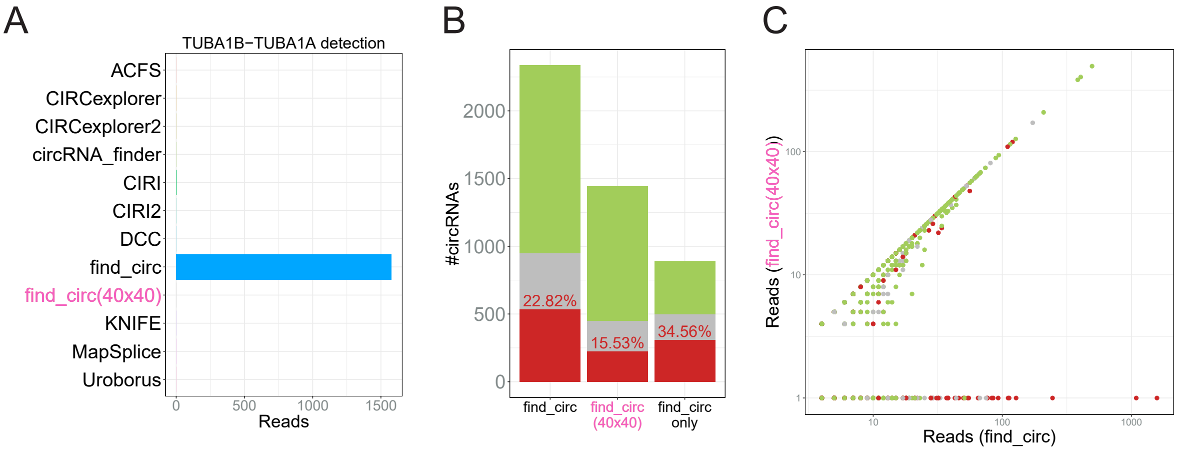

Supplementary Figure 2: Find_circ(40x40). A) Mis-detection of TUBA1A-TUBA1B circRNA using all algorithms including the stringent find_circ(40x40) with conservative threshold for mapping quality. **B)** Stacked barplot color-coded as Fig 1A comparing the fractions of true and false-positive, i.e. RNAseR resistant and RNAseR sensitive species, respectively, predicted by find_circ or find_circ(40x40). In addition, the subset of circRNA eliminated with increased threshold for mapping quality (find_circ only) is shown. **C)** Scatterplot on circRNAs predicted by default find_circ and find_circ(40x40) color-coded as in Fig 1A.

Supplementary Fig 3: False Negatives

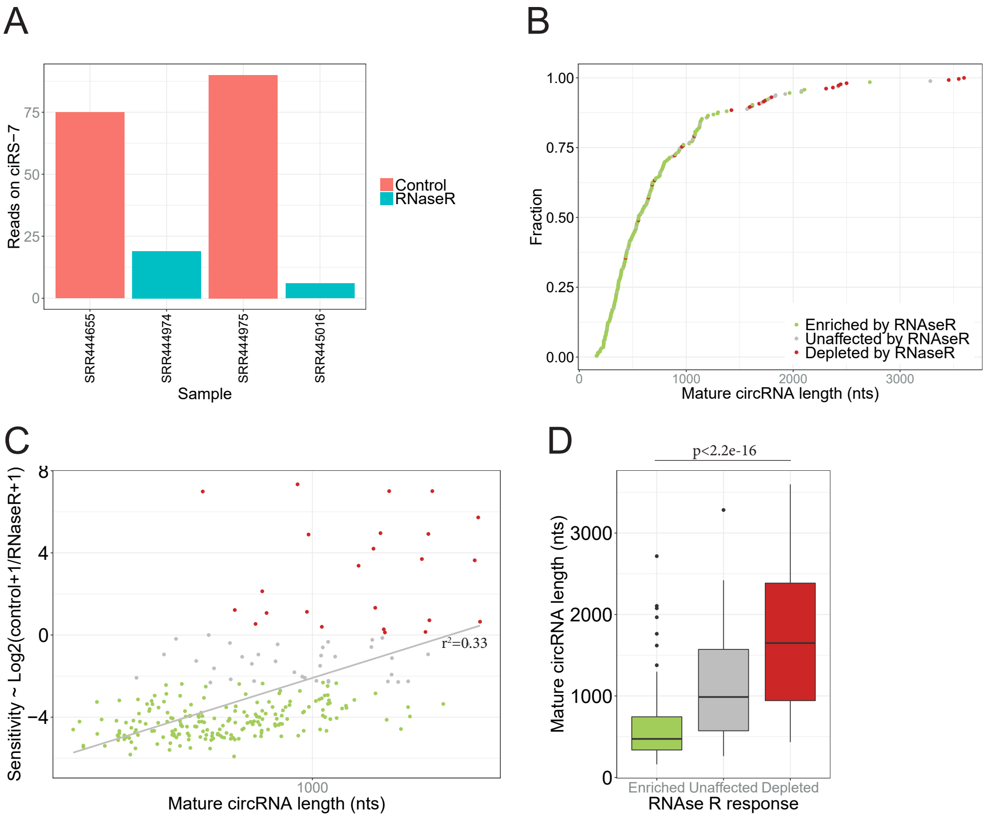

Supplementary Figure 3: False Negatives. A) Barplot with backsplice-spanning reads on ciRS-7 in each sample predicted by CIRI2. **B)** Cumulative fraction plot on mature circRNA length (i.e. the fully spliced circRNA according to hostgene annotations) on the subset of circRNAs predicted by all algorithms (n=259) color-coded as shown. **C)** Scatterplot on RNase R sensitivity as a function of mature circRNA length color-coded as in B. **D)** Boxplot on mature circRNA length subgroup by RNaseR sensitivity as shown. P-value is determined by Wilcoxon rank-sum test.

Supplementary Fig 4:

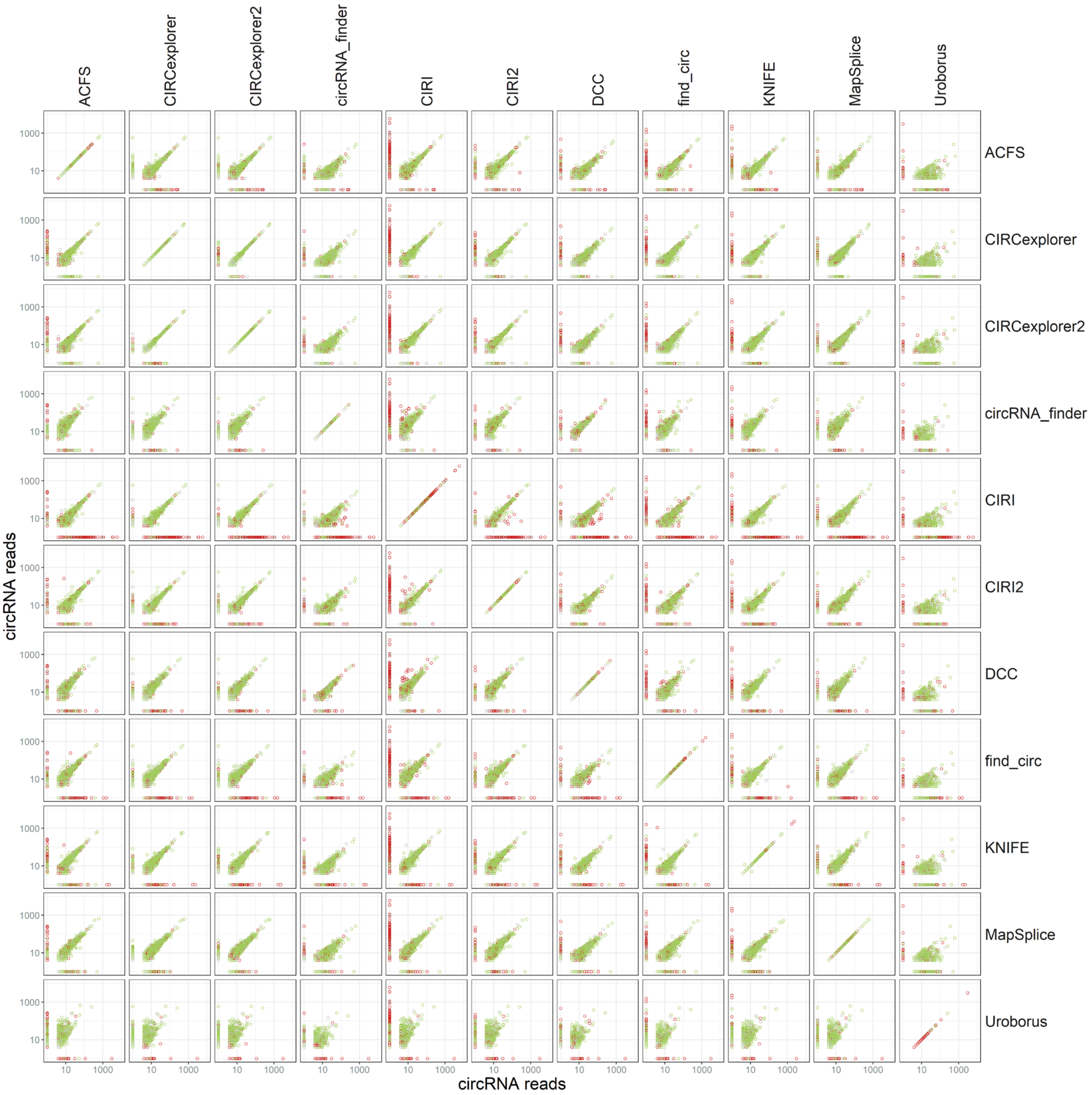

Supplementary Figure 4: Comparing algorithms. Scatterplots on circRNAs predicted by any two algorithms color-coded as in Fig. 1A. These individual scatterplots are interactively available at www.ncrnalab.dk/battle_of_algorithms.

Supplementary Fig 5:

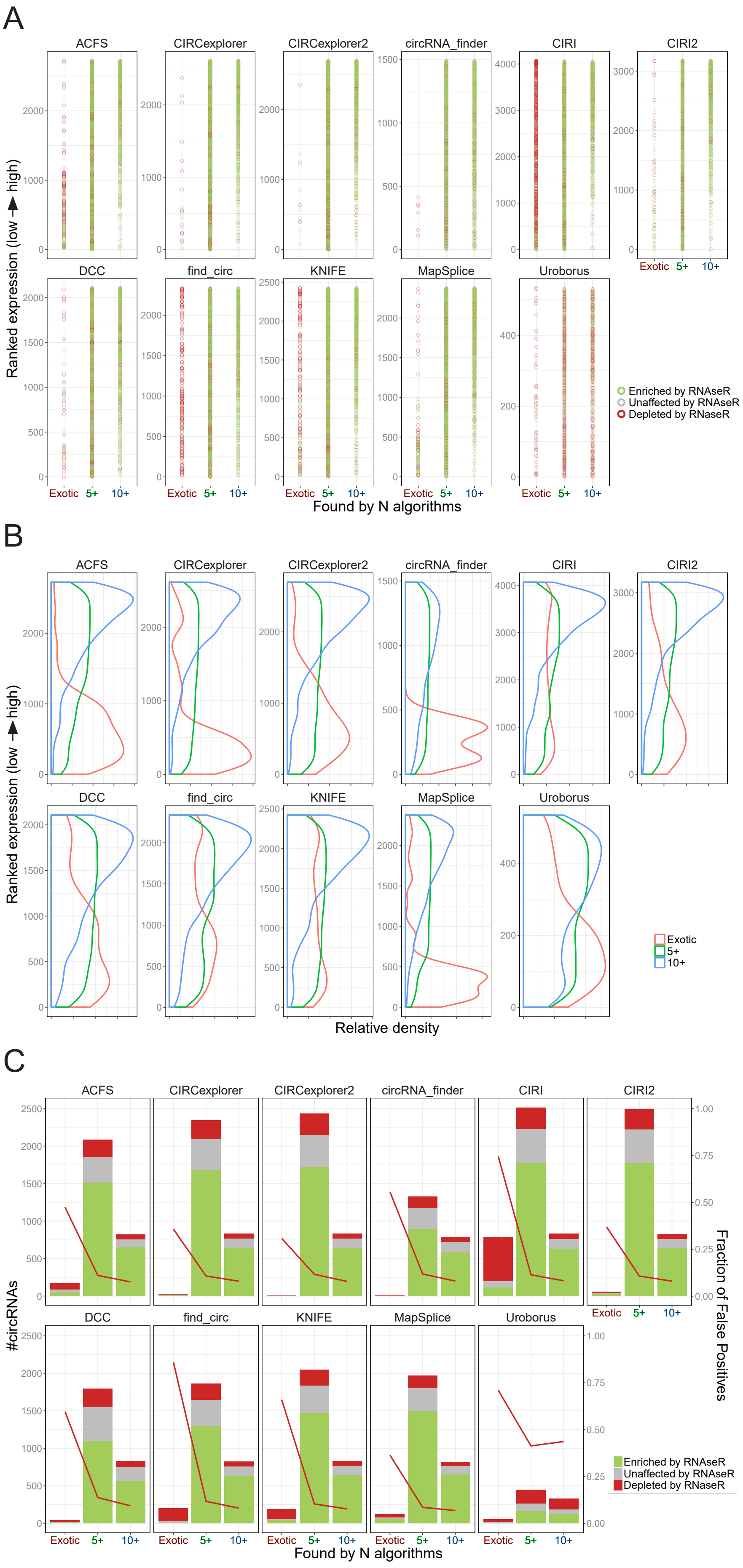

Supplementary Figure 5: circRNAs found by multiple algorithms. A) Ranked expression of circRNAs found exotically (only by one algorithm as denoted in strip), shared by at least 5 algorithms (5+) or shared by at least 10 algorithms (10+) color-coded as depicted. **B)** The density of circRNAs ranked by expression and subgrouped as in A. **C)** Number of predicted circRNAs subgrouped as in A and demarcated by RNaseR sensitivity (as in Fig 1A). Also, the fraction of false positives, i.e. RNAseR sensitive species, is depicted for each algorithm by red line (right-bound y-axis).

Supplementary Fig 6:

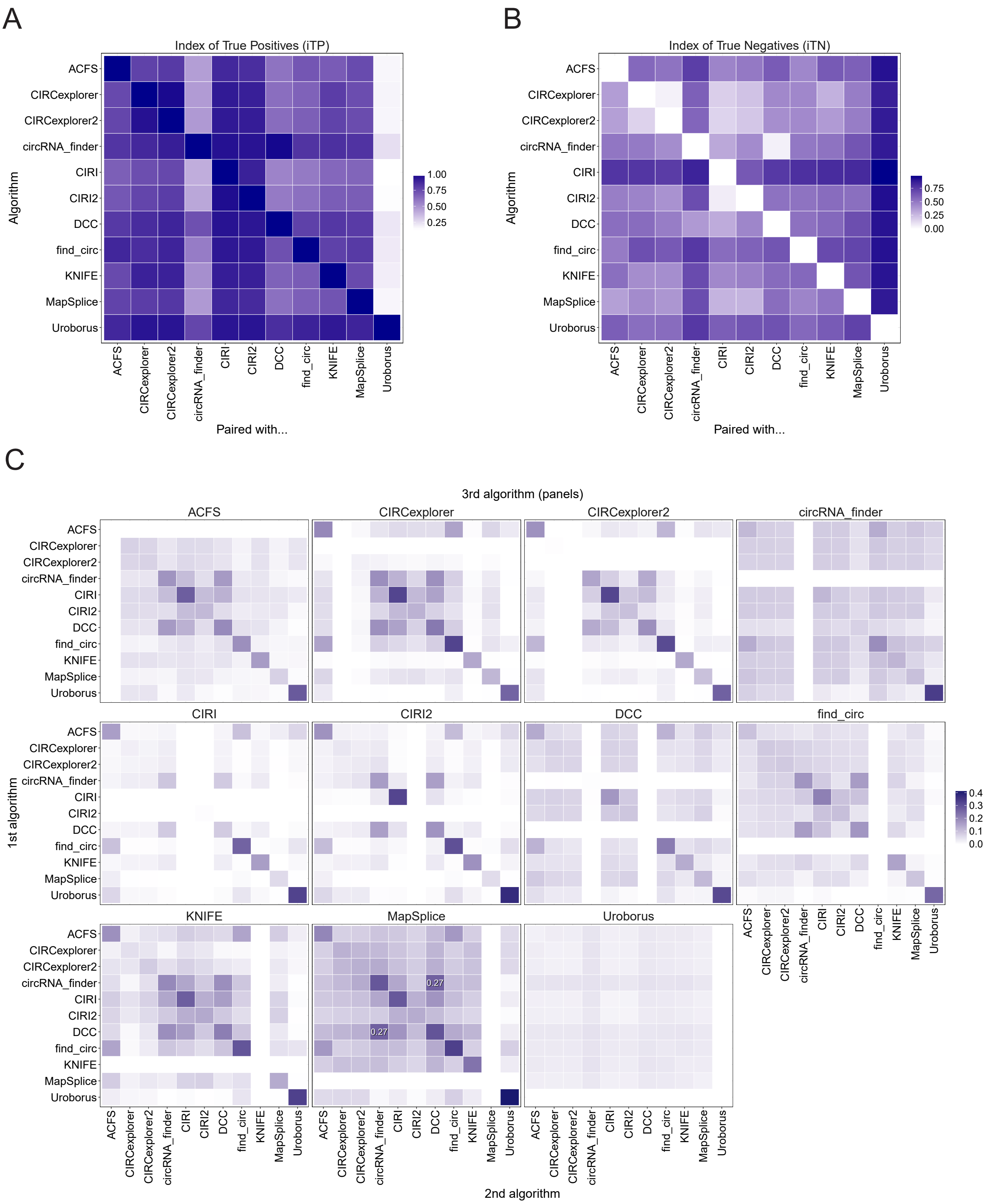

Supplementary Figure 6: Index of true positives and negatives. A-B) Heatmaps on iTP (true positive index, A) and iTN (true negative index, B). The true positive index (iTP) is defined as the fraction of true positive circRNAs, i.e. RNAseR resistant species, predicted by algorithm on y-axis and shared with algorithm on x-axis. The true negative index (iTN) is defined as 1-fN where fN is the fraction of RNaseR sensitive species predicted in algorithm denoted on y-axis and conjointly identified in other algorithm (x-axis). **C)** Three-wise Complementary scores. Here, the shared output from two algorithms (x and y-axis) was merged with a third algorithm, and the Complementary score was calculated as for the pair-wise comparison.

Supplementary Fig 7:

A

Accession	Samples	Reference	Total Reads
SRR1049826	HeLa - control#1	Mercer *et al*, 2015	152812658
SRR1049828	K562 - control#1	Mercer *et al*, 2015	189771806
SRR1049830	HeLa - RNAseR#1	Mercer *et al*, 2015	76442016
SRR1049832	K562 - RNAseR#1	Mercer *et al*, 2015	156983994

B

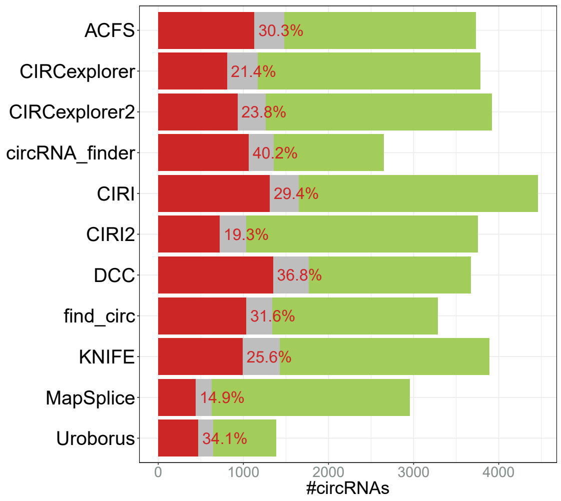

C

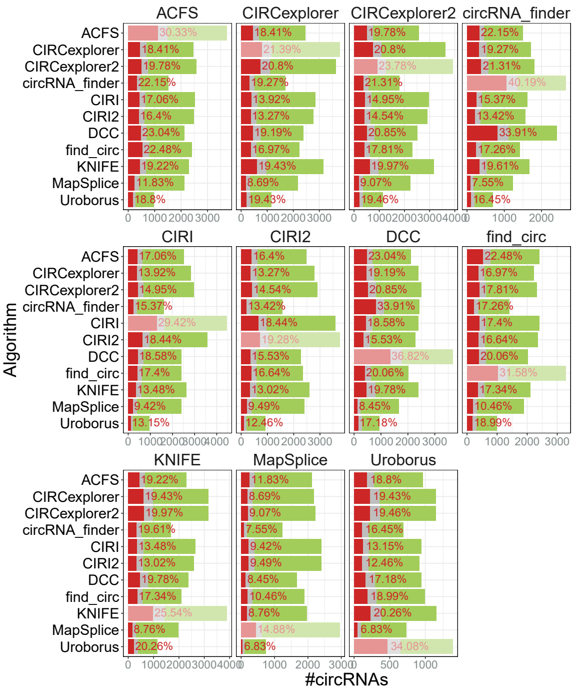

D

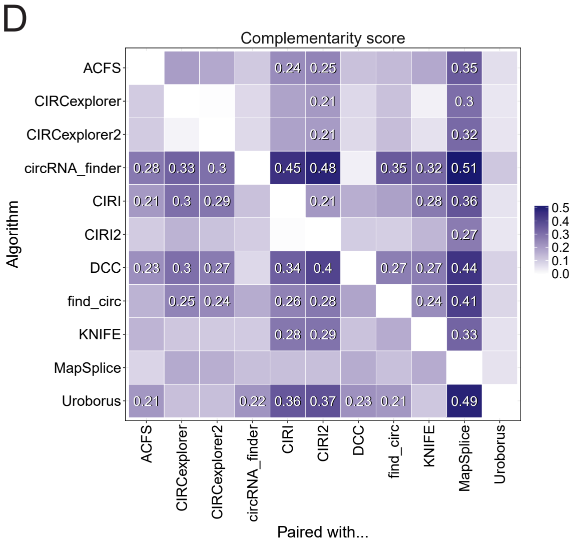

Supplementary Figure 7: CircRNA prediction on samples from GSE53327. A) Table with accession numbers, sample content and corresponding read counts. **B)** Stacked barplot (as in Fig 1A) of all predicted circRNAs stratified by RNAse R resistant (>= 2 fold enrichment, green), Unaffected (0.7-2 fold enrichment, grey) and RNAse R sensitive (below 0.7-fold enrichment in RNaseR treated samples, red), as denoted. Percentage reflects the fraction of RNaseR sensitive circRNAs defined as false positives. **C)** Comprehensive stacked barplot analysis of RNaseR sensitivity in the shared predictions by any two algorithms (as in Fig 4A). The 'dimmed' bars denote the unpaired algorithm. **D)** Heatmap on complementarity score (as in Fig 4C). The complementary score is calculated as (iTFxiTN)^2, where iTF is the fraction of true positive circRNAs (RNaseR resistant circRNAs, defined as in B) found in algorithm denoted on the y-axis and shared with algorithm on x-axis, and iTN is 1-fN, where fN is the fraction of RNaseR sensitive species conjointly identified in other algorithm. Complementary scores >= 0.2 are denoted specifically.

Supplementary Table 1: Exotic and abundant false positives

	circRNA coordinate			Reads (SRR44xxxx)			
**Tool**	**Chromosome**	**Start**	**End**	**4655**	**4974**	**4975**	**5016**
CIRI2	chrY	10035908	10037910	0	0	1016175	0
CIRI	chrY	10037756	10037910	548571	7479	0	5518
CIRI	chr17	33134804	33478372	277480	0	0	0
CIRI	chr19	36066504	36066612	18906	30292	32188	15442
CIRI	chr2	133012802	133012899	0	3255	5920	2390
CIRI	chr5	71146746	71146931	575	0	2990	0
CIRI	chr2	230045488	230045596	0	0	3457	0
Uroborus	chrUn_gl000220	155996	156152	3088	0	0	0
KNIFE	chr1	91852900	91852950	305	0	2008	828
KNIFE	chr11	85195100	85195150	238	0	1464	0
find_circ	chr12	49525080	49580616	476	235	1102	90
CIRI	chr16	33963109	33963327	1092	0	0	0
find_circ	chr12	49523282	49580241	274	107	816	55
CIRI	chr2	230045488	230045625	1054	0	0	0

Control
RNaseR

Supplementary Table 1: Exotic and abundant false positives. The genomic coordinates (hg19), responsible algorithm, and the associated read-counts for the 14 most highly abundant circRNA across all pipelines.
